# Book Notices

**Published:** 1868-03-01

**Authors:** 


### *BOOK NOTICES.*

Observations on the Nature and Treatment of Polypus of the Ear. By Edward H. Clark, M.D., Prof., etc. Boston: Ticknor & Fields.

This is a pamphlet of seventy-one pages, most artistically gotten up on tinted paper, and illustrated by two plates of thirteen figures. It is divided into two parts; part first consisting of a record and analysis of twelve cases; part second entering into an elaborate "description of polypoid growths of the ear, and their treatment, based chiefly on the preceding cases."

